# Is Cadmium Genotoxicity Due to the Induction of Redox Stress and Inflammation? A Systematic Review

**DOI:** 10.3390/antiox13080932

**Published:** 2024-08-01

**Authors:** Khulud Badawi, Basma M. El Sharazly, Ola Negm, Raheela Khan, Wayne G. Carter

**Affiliations:** 1 Clinical Toxicology Research Group, School of Medicine, University of Nottingham, Royal Derby Hospital Centre, Uttoxeter Road, Derby DE22 3DT, UK; khulud.badawi@nottingham.ac.uk (K.B.); basma.mohamed@med.tanta.edu.eg (B.M.E.S.); 2 Department of Laboratory Medicine, College of Applied Medical Sciences, Umm Al-Qura University, Makkah 24382, Saudi Arabia; 3 Parasitology Department, Faculty of Medicine, Tanta University, Tanta 31527, Egypt; 4 School of Medicine, University of Nottingham, Royal Derby Hospital Centre, Uttoxeter Road, Derby DE22 3DT, UK; ola.negm@nottingham.ac.uk (O.N.); raheela.khan@nottingham.ac.uk (R.K.); 5 Medical Microbiology and Immunology Department, Faculty of Medicine, Mansoura University, El-Mansoura 35516, Egypt

**Keywords:** cadmium, carcinogenicity, genotoxicity, inflammation, redox stress

## Abstract

The transition metal cadmium (Cd) is toxic to humans and can induce cellular redox stress and inflammation. Cd is a recognized carcinogen, but the molecular mechanisms associated with its genotoxicity and carcinogenicity are not defined. Therefore, a systematic review was undertaken to examine the scientific literature that has covered the molecular mechanism of Cd genotoxicity and its relationship to cellular redox stress and inflammation. An electronic database search of PubMed, Scopus, and the Web of Science Core Collection was conducted to retrieve the studies that had investigated if Cd genotoxicity was directly linked to the induction of redox stress and inflammation. Studies included exposure to Cd via in vitro and in vivo routes of administration. Of 214 publications retrieved, 10 met the inclusion criteria for this review. Preclinical studies indicate that Cd exposure causes the induction of reactive oxygen species (ROS) and, via concomitant activity of the transcription factor NF-κβ, induces the production of pro-inflammatory cytokines and a cytokine profile consistent with the induction of an allergic response. There is limited information regarding the impact of Cd on cellular signal transduction pathways, and the relationship of this to genotoxicity is still inconclusive. Nevertheless, pre-incubation with the antioxidants, N-acetylcysteine or sulforaphane, or the necroptosis inhibitor, necrostatin-1, reduces Cd toxicity; indicative that these agents may be a beneficial treatment adjunct in cases of Cd poisoning. Collectively, this review highlights that Cd-induced toxicity and associated tissue pathology, and ultimately the carcinogenic potential of Cd, may be driven by redox stress and inflammatory mechanisms.

## 1. Introduction

Cadmium (Cd) is a hazardous, non-essential transition metal that the National Toxicology Program and World Health Organization (WHO) recognize as a human carcinogen [1,2]. Cd is a known environmental pollutant that is a public health concern [3,4]. Humans can encounter Cd primarily through ingestion but also direct exposure via the respiratory tract, particularly through smoking. The Joint FAO/WHO Expert Committee on Food Additives (JECFA) recommends a Provisional Tolerable Monthly Intake (PTMI) limit of 25 µg/kg body weight for Cd [3,5].

Anthropogenic sources such as mining, sewage irrigation, and fertilizer applications, as well as atmospheric depositions, contribute to the introduction of Cd into soil and groundwater [6,7,8]. The consumption of foods including seafood, vegetables, and cereal-based products, inhalation of cigarette smoke, inappropriate handling of Cd itself, and drinking of Cd-infected water are all potential routes of human exposure. Cd exposure can also occur indirectly through the digestion of plants since Cd is absorbed by plants from soil polluted by Cd-enriched fertilizers and Cd-containing irrigation water [7,8,9]. Dietary exposure assessments indicate that the mean intake of Cd ranges from 0.6 µg/kg body weight per month (2.4% of the PTMI) for adults in the Sikasso region of Mali to 24 μg/kg body weight per month (96% of the PTMI) in children aged 4–11 years in China [5]. Although high percentile estimates of adult dietary Cd exposure occasionally exceed the PTMI, they typically range from 20 to 60% of the PTMI. The current PTMI for Cd is established based on its long half-life and the potential for bioaccumulation in tissues, particularly the kidneys; with a steady state not reached until after 45–60 years of exposure. While dietary exposure above the PTMI for limited periods might be of less concern in younger age groups, it could raise health concerns in areas where the adult Cd exposure exceeds the PTMI [5]. 

The exceptionally long biological half-life of Cd of 15–20 years renders it a potentially cumulative toxic agent and carcinogen [3,10]. Cd is sequestered in the liver and kidneys and may also replace calcium in bones, resulting in a variety of health problems such as kidney damage, renal dysfunction, hepatotoxicity, osteoporosis, and osteomalacia, as well as having a potential role in genotoxicity and malignancies [11,12]. 

Despite the fact that Cd is a classified carcinogen [1,2], the mechanisms associated with carcinogenesis remain unclear. There is an association of Cd with increased cancer risk and a negative impact on the immune system [12,13,14]. Cd exposure can increase the expression of certain inflammatory mediators and markers, as well as alter immune responses [14,15]. Cd exposure in micromolar concentrations results in the activation of signaling pathways that culminate with transcription factor activity, particularly NF-κβ and AP-1 in immune cells, as well as the upregulation of inflammatory markers and mediators [12,16]. Cd^2+^ can also substitute for Zn^2+^ in zinc finger proteins to exhibit transcriptional and translational effects [17]. 

Although carcinogenesis is a multistage process, it is conceptual to separate Cd-induced carcinogenesis into exposure of untransformed cells to Cd, and then a subsequent transformation to tumorigenic cells [18]. Although Cd is a bivalent cation and cannot directly generate damaging free radicals, Cd has the capacity to impact carcinogenesis due to its tendency to disrupt cellular homeostasis and the activity of second messengers, affecting the production of reactive oxygen species (ROS) and Ca^2+^ and inducing redox stress, and potentially lead to overexpression or enhanced activity of proto-oncogenes mediated in part by epigenetic effects [12,18,19,20,21,22]. 

Oxidative stress is characterized by a shift in the cellular redox balance in favor of pro-oxidants, which can influence signal transduction pathways and induce oxidative damage to cellular macromolecules including proteins, produce membrane lipid peroxidation, and induce genotoxicity through cross-links and breakages of DNA [23]. Studies have suggested that Cd-induced toxicity and carcinogenesis are linked to the ability of Cd to induce ROS [24,25,26,27,28,29]. However, there is still no proven link between ROS and Cd-induced carcinogenesis. Although Cd is a redox-inactive metal that does not catalyze any Fenton-type reactions, metals can generate oxidative stress by interacting with cellular reductants or suppressing the efficacy of cellular antioxidants, and Cd can generate hydroxyl radicals (•OH), a superoxide anion (O_2_•−), nitric oxide (NO), and hydrogen peroxide (H_2_O_2_) [26,27,28,30]. Hence, the aim of this systematic review was to review the evidence as to whether Cd genotoxicity is directly related to the Cd-induction of redox stress and inflammation.

## 2. Materials and Methods

### 2.1. Database Search

A systematic review was performed in accordance with the Preferred Reporting Items for Systematic Review (PRISMA) guidelines [31]. The PubMed, Scopus, and Web of Science electronic databases were searched from inception to February 2024 for the publications investigating whether the genotoxicity of Cd may be due to the induction of redox stress and inflammation based on the use of the keywords, “Cadmium”, “Cancerogenic”, and “Inflammation”. The search strategy and search terms used, together with the Medical Subject Headings (MeSH) combinations, are presented in Supplementary Table S1. In addition, references of relevant articles were manually searched for further relevant articles.

### 2.2. Study Selection

All references were stored in Ryann for subsequent screening against the pre-defined eligibility criteria and for the removal of duplicates (refer to Supplementary Table S2 for the PICO (population, inclusion, comparators, outcome) and inclusion and exclusion criteria). Eligible studies were included if they were published in English, involved cell culture (in vitro) or animal models (in vivo), and described a direct link between Cd exposure and carcinogenicity as the main study outcome evaluated by any method, such that Cd was reported as the intervention at any dosage or for any duration, and the study plan was experimental. Studies were excluded if they were not in English, involved human clinical trials, described a main outcome other than ROS generation or the induction of an inflammatory response, considered interventions related to other heavy metals, or the study design was non-experimental. The full texts of eligible studies were subsequently reviewed before a final analysis was conducted. 

### 2.3. Data Extraction and Management

The extraction of data and all searches were performed based on the defined eligibility criteria. All manuscripts that described Cd exposure and the induction of DNA damage and an immune response via ROS-dependent mechanisms in vitro were included, and the evidence was summarized into three tables. The first tabulated data include the studies that related to redox stress providing information on model species, concentrations of Cd administered, antioxidant enzymes (superoxide dismutase (SOD) and catalase (CAT), and lipid peroxidation. The second table covers typical inflammatory responses, including cytokine production, after Cd exposures. The third table includes characteristics of in vitro studies that considered antagonistic agents to combat Cd toxicity. This included details such as the model used, Cd concentrations employed, duration of exposure, examination of cell death, and assessment of ROS generation and comparison to control groups. Publications selected for data extraction were reviewed by the first author (KB) and the last author (WGC).

### 2.4. Quality and Risk of Bias Assessment

All eligible studies were assessed for quality and risk of bias (refer to Supplementary Table S3 and Supplementary Figure S1). In relation to the methodological quality assessment, data that covered the following were extracted from each study: randomization method adopted, study duration, random sequence generation, allocation concealment and blinding, as well as other bias-related risks based on information pertaining to reporting and attrition bias. Assessment of the risk of bias was performed using the SYRCLE tool [32]. The scoring system was as follows: ‘yes’ was selected if the risk of bias was reported; ‘no’ was chosen when unreported; and unclear or unknown denoted an ‘unclear’ score. Due to the heterogeneity of the studies in terms of design, methodology, and interventions used, a meta-analysis was not deemed feasible for this systematic review. 

## 3. Results

### 3.1. Search Findings

**Figure 1:**
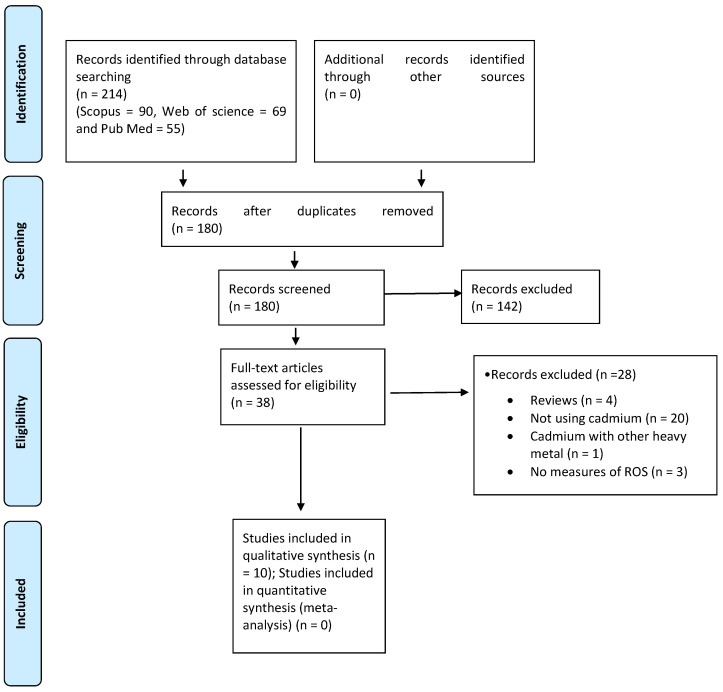
Preferred reporting items for systematic reviews and meta-analyses (PRISMA) flow chart [31].

The identification of eligible studies for this systematic review is illustrated in Figure 1. A total of 214 articles were identified through searches of the PubMed, Scopus, and Web of Science electronic databases. Following the removal of duplicates, 180 articles remained. A further 142 articles were subsequently excluded because they did not meet the inclusion criteria. The full texts of 38 potentially relevant studies were evaluated, leading to 10 eligible experimental studies selected for final analysis. The other 28 articles were excluded due to either not fulfilling the inclusion criteria or reporting irrelevant outcomes.

### 3.2. Characteristics of Eligible Studies

A summary of the characteristics of the 10 fully evaluated studies are outlined in Table 1 and Table 2, covering the in vivo and/or in vitro studies in relation to the induction of redox stress and an inflammatory response. Table 3 covers the characteristics of the in vitro studies of specific agents able to inhibit the toxic effects of Cd. Four studies used a zebrafish (AB strain), two used fish models (Cyprinus carpio), one utilized human gallbladder epithelial (GBP-9) cells, one study was undertaken with chicken peritoneal macrophage cells, one with a mouse Leydig (TM3) cell line, and one research group used two types of models, namely IPEC-J2 cells and swine small intestine. Four studies considered the ability of chemical agents (N-acetylcysteine (NAC), sulforaphane (SFN), and necrostatin-1 (NEC-1) to inhibit the toxic effects of Cd [33,34,35,36,37,38,39,40,41,42]. The dosage used varied in each study and the duration of Cd exposure was from 0 to 96 h. 

## 4. Discussion

Cd is a heavy metal that is hazardous to the health and is an environmental pollutant. This review was conducted to examine whether a systematic approach could provide a useful insight into the toxicity of Cd and its potential to induce carcinogenesis. Although published research has considered the mechanism of Cd toxicity, there is limited information that has specifically addressed the consequences of Cd-induced ROS and its associated cellular damage and Cd-induced inflammation. Collectively, the collated evidence covered in this review from both cell-based (in vitro) and animal (in vivo) studies suggests that oxidative stress promotes the activation of signaling pathways, resulting in pro-inflammatory cytokine production and inflammation, thereby potentially contributing to the cellular changes associated with a transformed phenotype.

### Cadmium Effects on Cellular Redox Stress and Inflammation

Increased ROS levels was a commonly encountered response to Cd [33,34,35,36,37,38,39,40,41,42] and this could damage DNA with genotoxic effects [12,18,19,20,43]. These damaging and potentially deleterious effects of excessive ROS production were countered by incubations with either the plant-derived antioxidant N-acetylcysteine (NAC), or sulforaphane (SFN), a potent organic sulfurous antioxidant, or necrostatin-1 (NEC-1), a necroptosis inhibitor, since these agents reduced ROS levels and the associated induction of apoptosis [36,39,40,41]. 

Cellular antioxidant defense is maintained in part by the activities of superoxide dismutase (SOD) and catalase (CAT). SOD is able to combat cellular oxidative stress due to its ability to scavenge oxygen radicals and decompose O_2_•− into hydrogen peroxide (H_2_O_2_) with the release of molecular oxygen, and CAT enzymes primarily catalyze the decomposition of H_2_O_2_ to water and molecular oxygen, collectively reducing the formation of certain ROS [44,45]. Similarly, the gene expression and protein activity of SOD activity was increased after exposure to Cd at non-lethal concentrations with levels that related to Cd exposure time (from 24 h to 96 h) [33]. Acute Cd exposure also increased levels of the Cu/Zn-SOD in the brain of zebrafish and CAT protein and activity but had no effect on mRNA levels [34]. By contrast, there was a downregulation of Cu/Zn-SOD and CAT mRNA, protein, and activity levels observed in the liver in response to Cd that correlated with the duration of Cd exposure [34]. Within the ovaries, Cd significantly raised Cu/Zn-SOD protein levels at 96 h but had no effect on the protein or activity levels at 24 h or mRNA levels at 96 h, whereas after 96 h of Cd exposure, the levels of CATs mRNA, protein, and activity decreased in the ovary [34].

The brain tissue of zebrafish that was acutely exposed to Cd at ZT0 (time at which the light intensity began to reach maximum) and at ZT12 (light intensity began to reach minimum) for 12 h had differential effects on antioxidant activities. CAT protein and activity was upregulated at ZT0 but there was downregulated mRNA, protein, and activity levels of CAT and Cu/Zn-SOD at ZT12 [35].

A product of redox stress is the oxidation of unsaturated lipids, typically in membranes, collectively termed lipid peroxidation. Lipid hydroperoxides (LOOH) are one of the principal products, and aldehydes such as malondialdehyde (MDA) and 4-hydroxynonenal (4-HNE) may also be generated as secondary products [46]. Hence, MDA is often used as a marker of the severity of lipid damage produced by free radicals. After Cd treatment, MDA levels in the brain and liver increased in response to the duration of Cd exposure [34]. However, contrary to the effect in the brain and liver, Cd exposure showed no significant effect on the levels of MDA in the ovary, indicative of a tissue-specific effect [34]. Similarly, Cd treatment had temporal effects on immunity; fish exposed at two separate times (ZT0 and ZT12) elicited different immunological responses [35], so the ability of tissues to uptake Cd and the potential for bioaccumulation within tissues is tissue-specific and related to Cd exposure concentration [47].

Lymphocytes extracted from the pronephros and spleens of carp had higher levels of MDA after treatment of the cells with a low concentration of Cd [40]. Excessive Cd to zebrafish also increased MDA content [33]. The antioxidant NAC reduced the release of free radicals and improved the oxidative stress induced by Cd in swine and this was evidenced by a reduction in MDA levels [41]. 

Immune cells can be damaged by oxidative stress and ROS accumulation, and this could potentially weaken immune defense. There was a significant upregulation of the pro-inflammatory cytokines TNF-α, IL1-β, IFN-γ, and IL-8 in the Cd-treatment groups compared to the matched controls, consistent with Cd induction of inflammation [33]. Similarly, Cd immunotoxicity was evident from elevated TNF-α protein levels following Cd exposure within brain, liver, and ovary tissue of zebrafish [34]. Upregulated mRNA levels of NF-κβ were concomitant with TNF-α expression levels in the liver [34], indicative of the interplay between these pro-inflammatory genes, and the role of active nuclear NF-κβ as a driver for pro-inflammatory gene expression. However, a discrepancy in the correlation between NF-κβ and the expression of its target genes was noted in both the brain and ovaries. Specifically, in the brain, the levels of COX-2 and iNOS expression increased while NF-κβ expression remained unaltered. Conversely, in the ovaries, mRNA levels of COX-2 and iNOS showed no significant changes, while NF-κβ expression levels were upregulated [34]. 

T-helper cells differentiate into two major subsets, Th1 and Th2, with distinct functions and cytokine profiles, and inflammation is frequently associated with a Th1/Th2 imbalance. Th1 cells produce IL-2, IFN-γ, and TNF-α, which are responsible for cell-mediated immunity and phagocyte-dependent inflammation [48,49]. Th2 cells produce IL-4, IL-5, IL-10, and IL-13, which induce strong antibody responses and eosinophil accumulation while inhibiting some phagocyte functions, and the production of IL-4 also provides some control of Th1 activity since it inhibits IFN-γ and IL-4 inhibits Th1 differentiation [49]. Cd exposure (to humans) can impact the Th1/Th2 balance by influencing Th cell sub-sets (Th1, Th2, Th17, Tregs) with the induction of a predominantly pro-inflammatory cytokine profile [50]. The inhibitory effects of Cd on the production of the Th1 signature cytokine IFN-γ and upregulation of the Th2 cytokine IL-4 (in response to Cd) may not only inhibit the generation of Th1 responses but could also potentially increase sensitivity to the development of allergies [49]. A dysregulated immune response as a consequence of Cd exposure can predispose individuals to the worsening of psoriasis [14], a condition in which Th1 and Th17 cells play crucial roles, and this highlights the connection between occupational exposure to Cd and immune dysfunction and inflammatory diseases [50].

Cd exposure stimulates neutrophils to release cytokines including IL-6, IL-10, IL-1β and IFN-γ [35]. IL-10 is often considered an anti-inflammatory cytokine and therefore the sharp rise in IL-10 levels might relate to the control of excessive inflammation and prevent tissue damage. Alternatively, the increase in IL-10 could reflect its (co)-stimulation with inflammation, which does occur in certain inflammatory conditions [51,52,53]. Other cytokine studies reported increased levels of IL-4, IL-6, and IL-10 in response to Cd, while IFN-γ decreased [41], suggesting a possible influence on Th1/Th2 immune balance. TNF-α, IL-1, and NF-κβ levels in the Cd group were also significantly higher than controls for swine intestine [41], and similar results were obtained in the Cd-treated IPEC-J2 cells [41].

## 5. Conclusions

**Figure 2:**
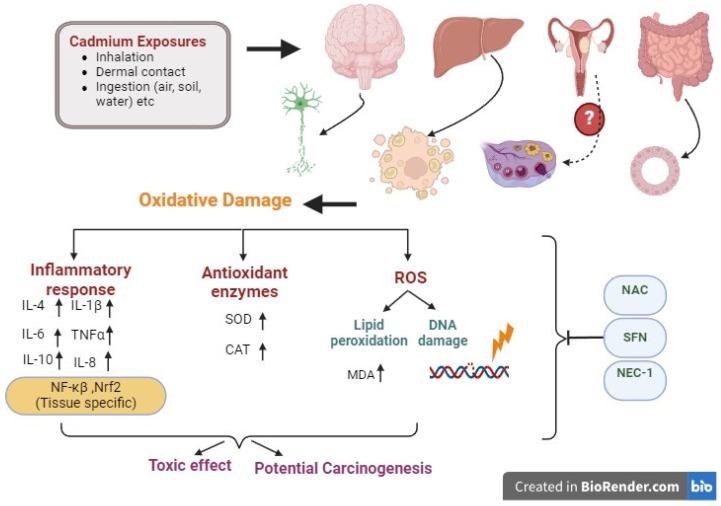
Summary of the effects of Cd exposure on redox stress and induction of an inflammatory response in different organs and tissues.

Although further studies are needed to improve the understanding of the toxicity of Cd and its linkage to carcinogenesis, oxidative stress and induction of inflammation are the identified risk factors. The adverse impact caused by ROS can trigger an imbalance in redox metabolism, which, in turn, induces a pro-inflammatory cytokine profile and promotes leukocyte cell recruitment. By investigating and clarifying these deleterious mechanisms, future therapies such as antioxidants and anti-inflammatory agents may be able to limit Cd promoter effects on tumorigenesis. Furthermore, this review has highlighted that the potential for Cd-induced carcinogenesis can be influenced by dosage, duration of exposure, penetration into the skin and, internalization by mammalian cells, as well as tissue-specific redox and antioxidant status and potential for inflammation (Figure 2). Indeed, ROS levels and associated damage such as lipid peroxidation are observed in some but not all chronic studies of Cd dosing [54], indicative of cellular adaptation such as the upregulation of the non-enzymatic and enzymatic antioxidant defense systems, and the potential for reduced Cd due to sequestration by metallothioneins [55]. 

Ultimately, the induction of ROS and a pro-inflammatory milieu provides a basis, at least acutely, for cellular and DNA damage and an impact on cellular signaling that could promote cellular transformation. Furthermore, presumably, the Cd-induced genomic damage and cellular instability need to be below the threshold of induction of apoptosis and cell death to facilitate long-lasting, pro-tumorigenic, and cumulative damage, but this will be difficult to prove equivocally for chronic Cd exposures.

The primary routes of exposure to Cd are inhalation, dermal contact, and ingestion through air, soil, sediment, and water. The molecular processes that lead to Cd-induced oxidative stress in mammalian organs are not fully delineated, but the engagement of cellular mitochondria is likely, given their critical role in the production of excess ROS. Cd disrupts antioxidant defense mechanisms (superoxide dismutase (SOD) and catalase (CAT) levels and activities). Collectively, this can result in lipid peroxidation, such as formation of MDA, and DNA damage. Cellular pre-treatment with antioxidants such as N-acetylcysteine (NAC) or sulforaphane (SFN) reduce Cd-induced oxidative stress. Similarly, necrostatin-1 (NEC-1), a necroptosis inhibitor, limits Cd toxicity and the induction of cell death. Cd induces increased levels of pro-inflammatory cytokines, IL-1β, IL-4, IL-6, TNF-α, and IL-8, as well as IL-10, the latter of which is often anti-inflammatory. Cd-induction of oxidative mechanisms are time-dependent and tissue-specific, including the induction of NF-κβ and Nrf2, and contribute to an inflammatory response. Hence, exposure to Cd has the potential to cause both acute toxic effects as well as potential carcinogenesis from sustained exposures.

## Figures and Tables

**Table 1:** Studies that considered redox stress in response to cadmium exposures.

Author	Model	Cd Dosing Regimen	ROS Production	Antioxidant Enzymes		Lipid Peroxidation
				SOD Levels/ Activity	CAT Levels/ Activity	MDA
Yin et al., 2018 [33]	Zebrafish (AB strain)	Cd: 3 mg/L at 24, 48, 72 and 96 h	↑ at 24, 48, 72 and 96 h (*p* < 0.05)	↑ SOD1 gene expression at 48, 72 and 96 h (*p* < 0.05) ↑ SOD2 gene expression at 24, 48, 72 and 96 h (*p* < 0.05) ↑ SOD activity at 24, 84, 72 and 96 h (*p* < 0.05)	ND	↑ at 48, 72 and 96 h (*p* < 0.05)
Zheng et al., 2016 [34]	Zebrafish (AB strain)	Cd:1 mg/L at 24 and 96 h	Brain ↑ at 24 and 96 h (*p* = 0.05)	Brain ↑ protein and activity levels of Cu/Zn-SOD at 24 and 96 h (*p* = 0.05) mRNA levels of Cu/Zn-SOD: No change at 24 and 96 h	Brain mRNA levels: no change ↑ protein and activity levels (*p* = 0.05)	Brain ↑ at 96 h (*p* = 0.05)
			Liver ↑ at 96 h (*p* = 0.05)	Liver ↑ mRNA levels of Cu/ZnSOD at 24 and 96 h (*p* = 0.05) ↓ protein and activity levels of Cu/Zn-SOD at 96 h (*p* = 0.05)	Liver ↓ mRNA and activity levels at 24 and 96 h (*p* = 0.05) protein levels no change at 24 and 96 h	Liver ↑ at 96 h (*p* = 0.05)
			Ovary No change at 24 and 96 h	Ovary ↑ mRNA levels of Cu/ZnSOD at 24 h ↑ protein levels of Cu/Zn-SOD at 96 h (*p* = 0.05), no change in activity levels at 24 and 96 h	Ovary ↑ mRNA, protein and activity levels at 96 h (*p* = 0.05)	Ovary No change
Zheng et al., 2017 [35]	Zebrafish (AB strain)	Cd: 0.97 mg Cd L^−1^	↑ ZT0 ↑ Z12 (*p* < 0.05)	activity and protein levels of Cu/Zn-SOD ZT0 vs. ZT12: no change ↓ mRNA levels of Cu/Zn-SOD ZT12 vs. ZT0 (*p* < 0.05)	mRNA levels ZT0 vs. ZT12: no change ↓ activity and protein levels at ZT12 vs. ZT0 (*p* < 0.05)	ND
Sharma et al., 2020 [36]	Human gallbladder epithelial cells (GBP-9)	Cd: 2.5, 5, and 10 µM	↑ (*p* < 0.05)	ND	ND	ND
Zheng et al., 2020 [37]	Carp lymphocytes	Cd: 40 µM 24 h	pronephros group ↑ (*p* < 0.05)	pronephros group ↓ T-SOD (*p* < 0.05)	pronephros group ↓ (*p* < 0.05)	pronephros group ↑ (*p* < 0.05)
			spleens group ↑ (*p* < 0.05)	spleens group ↓ T-SOD (*p* < 0.05)	spleens group ↓ (*p* < 0.05)	spleens group ↑ (*p* < 0.05)
Jiaxn et al., 2020 [38]	Carp neutrophils	Cd:10 µM 2 h	↑ (*p* < 0.01)	↑ (*p* < 0.001)	↑ (*p* < 0.001)	ND
Yang et al., 2019 [39]	Mouse Leydig (TM3) cells	Cd: 10 µM	↑ (*p* < 0.01) SFN group: (2.5, 5, 10 µM) ↓ (*p* < 0.05)	↓ (*p* < 0.05) SFN group: ↑ (*p* < 0.05)	ND	↑ (*p* < 0.05) SFN group: ↓ (*p* < 0.05)
Zhang et al., 2020 [40]	Chicken peritoneal macrophages	Cd: 20 and 50 μM 12 h	↑ 20 and 50 μM Cd (*p* < 0.05)	ND	ND	ND
Chen et al., 2022 [41]	IPEC-J2 cells and swine small Intestine tissue	Cd: 5 µΜ NAC: 2.5 mM Nec-1: 20 mM 6 h 20 mg/kg Cd dosing for experimental animals for 40 days	↑ Cd group (*p* < 0.05) ↓ NAC group (*p* < 0.05) ↓ Cd + NAC group vs. Cd ↓ Cd + Nec-1 vs. Cd (*p* < 0.05)	↓ activity in Cd group (*p* < 0.05) ↑ Cd + NAC group vs. Cd (*p* < 0.05) ↑ Cd + Nec-1 group vs. Cd (*p* < 0.05)	ND	↑ Cd group (*p* < 0.05) ↓ Cd + NAC group vs. Control (*p* < 0.05) ↓ Cd + Nec-1 group vs. control (*p* < 0.05)
Yin et al., 2017 [42]	Adult Zebrafish (AB strain)	Cd:12 mg/L	↑ at 48, 72, and 96 hpf (*p* < 0.05)	↑ SOD1 and ↑ SOD2 mRNA expression at 24, 48, 72, and 96 hpf (*p* < 0.05)	↑ mRNA expression at 48, 72 and 96 hpf (*p* < 0.05)	ND

Abbreviations: CAT, Catalase; Cd, Cadmium; Cu/Zn-SOD, Copper/zinc superoxide dismutase; hpf, hours post-fertilization; IL-1β, Interleukin 1β; IL-6, Interleukin-6; IL-8, Interleukin-8; IPEC-J2 cells, intestinal porcine enterocytes isolated from the jejunum of a neonatal unsuckled piglet; mRNA, messenger ribonucleic acid; NAC, N-acetylcysteine; ND, not determined; Nec-1, necrostatin-1; NF-κβ, nuclear factor kappa-light-chain-enhancer of activated B cells; Nrf2, nuclear factor erythroid-2 related factor 2; ROS, reactive oxygen species; SFN, sulforaphane; SOD1, superoxide dismutase 1; SOD2, superoxide dismutase 2; TNF-α, Tumor necrosis factor-alpha; Z0, time at which the light intensity began to reach maximum; Z12, time at which the light intensity began to reach minimum.

**Table 2:** Studies that considered inflammation in response to cadmium exposures.

Author	Model	Cd Dosing Regimen	Inflammatory Signaling		
			Nrf2	NF-κβ	Cytokines
Yin et al., 2018 [33]	Zebrafish (AB strain)	Cd: 3 mg/L at 24, 48, 72 and 96 h	↑gene expression at 24, 48, 72 and 96 h (*p* < 0.05)	ND	↑ TNF-α at 24, 72 and 96 h (*p* < 0.05) ↑ IL-1β at 24, 48, 72 and 96 h (*p* < 0.05) ↑ IFN-γ at 24, 48, 72 and 96 h (*p* < 0.05) ↑ IL-8 at 24, 48, 72 and 96 h (*p* < 0.05)
Zheng et al., 2016 [34]	Zebrafish (AB strain)	Cd:1 mg/L at 24 and 96 h	Brain mRNA no change at 24 and 96 h	Brain mRNA no change at 24 and 96 h	Brain mRNA of TNF-α: No change ↑ protein level of TNF-α at 24 and 96 h (*p* = 0.05)
			Liver ↑ mRNA of Nrf2 at 24 h (*p* = 0.05) ↓ mRNA of Nrf2 at 96 h (*p* = 0.05)	Liver ↑ mRNA of NF-κβ at 24 h (*p* = 0.05) ↓ mRNA of NF-κβ at 96 h (*p* = 0.05)	Liver ↑ mRNA of TNF-α at 24 and 96 h ↑ protein level of TNF-α at 96 h (*p* = 0.05)
			Ovary ↑ mRNA of Nrf2 at 24 h (*p* = 0.05)	Ovary ↑ mRNA of NF-κβ at 24 h (*p* = 0.05)	Ovary mRNA of TNF-α: No change ↑ protein level of TNF-α at 96 h (*p* = 0.05)
Zheng et al., 2017 [35]	Zebrafish (AB strain)	Cd: 0.97 mg Cd L^−1^	↓ mRNA level of Nrf2: ZT12 vs. ZT0 (*p* < 0.05)	mRNA level of NF-κβ ZT0 vs. ZT12: no change	TNF-α mRNA level ZT0 vs. ZT12: no change ↓ protein level of TNF-α ZT12 vs. ZT0 (*p* < 0.05)
Sharma et al., 2020 [36]	Human gallbladder epithelial cells (GBP-9)	Cd: 2.5, 5, and 10 µM	ND	ND	ND
Zheng et al., 2020 [37]	Carp lymphocytes	Cd: 40 µM 24 h	ND	ND	pronephros group ↑ IL-1β mRNA and protein (*p* < 0.05) ↑ IL-6 mRNA and protein (*p* < 0.05)
			ND	ND	spleens group ↑ IL-1β mRNA and protein (*p* < 0.05) ↑ IL-6 mRNA and protein (*p* < 0.05)
Jiaxn et al., 2020 [38]	Carp neutrophils	Cd:10 µM 2 h	ND	ND	mRNA expression: ↑ TNF-α (*p* < 0.05) ↑ IL-1β (*p* < 0.01) ↑ INF-γ (*p* < 0. 01) ↑IL-10 (*p* < 0. 01) ↑ IL-6 (*p* < 0.001)
Yang et al., 2019 [39]	Mouse Leydig (TM3) cells	Cd: 10 µM	↑ mRNA and protein (*p* < 0.05) SFN group: ↓ (*p* < 0.05)	ND	ND
Zhang et al., 2020 [40]	Chicken peritoneal macrophages	Cd: 20 and 50 μM 12 h	ND	ND	mRNA expression: ↑ IL-1β ↑ IL-6 ↑ TNF-α (*p* < 0.05)
Chen et al., 2022 [41]	IPEC-J2 cells and swine small Intestine tissue	Cd: 5 µΜ NAC: 2.5 mM Nec-1: 20 mM 6 h 20 mg/kg Cd dosing for experimental animals for 40 days	ND	Cd group for swine small intestine, mRNA expression: ↑ NF-κβ (*p* < 0.05) Cd group for swine small intestine, protein levels: ↑ NF-κβ (*p* < 0.05) Cd group for IPEC-J2, mRNA expression: ↑ NF-κβ (*p* < 0.05) Cd group for IPEC-J2, protein levels: ↑ NF-κβ (*p* < 0.05) Cd + NAC and Cd + Nec-1 vs. Cd group IPEC-J2 cell mRNA expression: ↓ NF-κβ (*p* < 0.05) Cd + NAC and Cd + Nec-1 vs. Cd group IPEC-J2 cell protein expression: ↓ NF-κβ (*p* < 0.05)	Cd group for swine small intestine, mRNA expression: ↑TNF ↑ IL-1β ↑ IL-6 (*p* < 0.05) Cd group for swine small intestine, protein levels: ↑ TNF-α ↑ IL-1β ↓ IFN-γ ↑ IL-10 ↑ IL-4 (*p* < 0.05) Cd group for IPEC-J2, mRNA expression: ↑ TNF ↑ IL-1β ↑ IL-6 ↓ IFN-γ (*p* < 0.05) Cd group for IPEC-J2, protein levels: ↑ TNF-α ↑ IL-1β ↓ IFN-γ ↑ IL-10 ↑ IL-4 (*p* < 0.05) Cd + NAC and Cd + Nec-1 vs. Cd group IPEC-J2 cell mRNA expression: ↑ TNF ↓ IL-1β ↓ IL-6 ↑ IFN-γ (*p* < 0.05) Cd + NAC and Cd + Nec-1 vs. Cd group IPEC-J2 cell protein expression: ↓ TNF-α ↓ IL-1β ↑ IFN-γ ↓IL-10 ↓ IL-4 (*p* < 0.05)
Yin et al., 2017 [42]	Adult Zebrafish (AB strain)	Cd:12 mg/L	↑ mRNA expression at 48, 72, and 96 hpf (*p* < 0.05)	ND	mRNA expression: ↑ TNF-α at 72, and 96 hpf (*p* < 0.05) ↑ IL-1β at 24, 48, 72, and 96 hpf (*p* < 0.05) ↑ INF-γ at 48, 72, and 96 hpf (*p* < 0.05)

Abbreviations: Cd, Cadmium; hpf, hours post-fertilization; IL-1β, Interleukin 1β; IL-6, Interleukin-6; IL-8, Interleukin-8; IPEC-J2 cells, intestinal porcine enterocytes isolated from the jejunum of a neonatal unsuckled piglet; mRNA, messenger ribonucleic acid; NAC, N-acetylcysteine; ND, not determined; Nec-1, necrostatin-1; NF-κβ, nuclear factor kappa-light-chain-enhancer of activated B cells; Nrf2, nuclear factor erythroid-2 related factor 2; ROS, reactive oxygen species; SFN, sulforaphane; TNF-α, Tumor necrosis factor-alpha; Z0, time at which the light intensity began to reach maximum; Z12, time at which the light intensity began to reach minimum.

**Table 3:** Characteristics of the in vitro studies that considered antagonists of cadmium toxicity.

Author	Model	Concentration	Cell Death	ROS
Sharma et al., 2020 [36]	Human gallbladder (GBP-9) epithelial cells	0–10 µM NAC: 5 mM 2 h	↑ Cd group ↓ Pre-treatment 5 mM NAC + Cd group (*p* < 0.05)	↑ Cd group (0.5, 1, 2.5, 5, and 10 μM) pre-treatment with NAC and Cd group: ↓ (*p* < 0.05)
Yang et al., 2019 [39]	Mouse Leydig (TM3) cells	Cd:10 µM SFN: 2.5, 5, and 10 µM	↑ Cd group ↓ Cd + SFN group ↓ Cd group vs. Cd + SFN group (*p* < 0.05)	↑ (*p* < 0.01) ↑ SFN 2.5, 5, and 10 µM (*p* < 0.01) ↓ SFN + Cd groups vs. Cd group (*p* < 0.01 at 2.5 and 5 µM and *p* < 0.05 at 10 µM)
Zhang et al., 2020 [40]	Chicken peritoneal macrophages	Cd: 20 and 50 µM for 12 h NAC: 500 µM for 2 h	pre-treatment NAC alone: no change ↓ pre-treatment NAC with 20 and 50 µM Cd (*p* < 0.05)	↓ NAC group vs. the control group (*p* < 0.05) ↑ 20 and 50 μM Cd group (*p* < 0.05) ↓ Pre-treatment NAC + 20 or 50 μM Cd group (*p* < 0.05)
Chen et al., 2022 [41]	IPEC-J2 cells	Cd: 5 mM NAC: 2.5 mM Nec-1: 20 mM 6 h	↑ Cd group ↓ Cd + NAC ↓ Cd + Nec-1 (*p* < 0.05)	↑ Cd group ↓ Cd + NAC (*p* < 0.05) ↓ Cd + Nec-1 (*p* < 0.05)

Abbreviations: Cd, Cadmium; IPEC-J2 cells, intestinal porcine enterocytes isolated from the jejunum of a neonatal unsuckled piglet; NAC, N-acetylcysteine; Nec-1, necrostatin-1; SFN, sulforaphane.

## Data Availability

Data supporting the results are included as Supporting Data.

## Supplementary Materials

The following supporting information can be downloaded at: https://www.mdpi.com/article/10.3390/antiox13080932/s1, Supplementary Table S1. The medical databases and search terms used for the systematic review; Supplementary Table S2. PICO and inclusion and exclusion criteria for the systematic review; Supplementary Table S3. Risk of bias and assessment according to the SYRCLE criteria; Supplementary Figure S1. Risk of bias and assessment according to the SYRCLE criteria.
