# Supplementary material for: Is Cadmium Genotoxicity Due to the Induction of Redox Stress and Inflammation? A Systematic Review
Source: Antioxidants (Basel). 2024 Aug 1;13(8):932. doi: 10.3390/antiox13080932 (PMC11351676; doi:10.3390/antiox13080932)
Supplement: Supplementary file 1 [file antioxidants-13-00932-s001.zip › antioxidants-3074543-supplementary.pdf]

Supplementary Data

Supplementary Table S1. The medical databases and search terms used for the systematic review.

Data base	Search Strategy	Results
PubMed:<br><br>#1<br>Cadmium	(Cadmium [Title/Abstract])) OR (Cadmium [MeSH Terms])<br><br>English	12.268
#2<br>Cancerogenic	(Genotoxicity [Title/Abstract] OR ROS formation [MeSH Terms] OR Oxidative DNA [MeSH Terms] OR "Oxidative DNA "[Title/Abstract] OR "Free radical* "[Title/Abstract] OR "superoxide dismutase" [MeSH Terms] OR "redox stress "[Title/Abstract] OR "DNA damage "[Title/Abstract] OR " DNA damage "[MeSH Terms])	72.133
#3<br>Inflammatory response	(" Inflammatory response"[Title/Abstract] OR " Inflammatory signalling"[Title/Abstract] OR " NF-kB"[Title/Abstract] OR " pro-inflammatory cytokines "[Title/Abstract] OR " IL-6"[Title/Abstract] OR " IL-8 "[Title/Abstract])	78.692
#1AND #2AND #3	(Cadmium [MeSH Terms] OR Cadmium [Title/Abstract]) AND (genotoxicity[Title/Abstract] OR ROS formation [MeSH Terms] OR Oxidative DNA [MeSH Terms] OR "Oxidative DNA "[Title/Abstract] OR "Free radical* "[Title/Abstract] OR "superoxide dismutase" [MeSH Terms] OR "redox stress "[Title/Abstract] OR "DNA damage "[Title/Abstract]OR " DNA damage "[MeSH Terms])AND (" Inflammatory response"[Title/Abstract] OR " Inflammatory signalling"[Title/Abstract] OR " NF-kB"[Title/Abstract] OR " pro-inflammatory cytokines "[Title/Abstract] OR " IL-6"[Title/Abstract] OR " IL-8 "[Title/Abstract])<br><br>English	55
Scopus:<br><br>#1<br>Cadmium	TITLE-ABS-KEY (cadmium) AND (LIMIT-TO (DOCTYPE , "ar")) AND (LIMIT-TO (LANGUAGE , "English"))	171.712
#2		261.818

Cancerogenic	TITLE-ABS-KEY ((genotoxicity OR " ROS formation " OR "Oxidative DNA " OR "Free radical " OR "Free radical* " OR "superoxide dismutase" OR "redox stress " OR "DNA damage ")) AND (LIMIT-TO (EXACTKEYWORD , "Article")) AND (LIMIT-TO (LANGUAGE , "English"))	
#3 Inflammatory response	TITLE-ABS-KEY ((" Inflammatory response" OR " Inflammatory signalling" OR " NF-kB" OR " pro-inflammatory cytokines " OR " IL-6" OR " IL-8 "))) AND (LIMIT-TO (DOCTYPE , "ar")) AND (LIMIT-TO (LANGUAGE , "English"))	231.694
#1 AND #2 AND#3	TITLE-ABS-KEY (((cadmium)) AND ((genotoxicity OR " ROS formation " OR "Oxidative DNA " OR "Free radical " OR "Free radical* " OR "superoxide dismutase" OR "redox stress " OR "DNA damage ")) AND ((" Inflammatory response" OR " Inflammatory signalling" OR " NF-kB" OR " pro-inflammatory cytokines " OR " IL-6" OR " IL-8 "))) AND (LIMIT-TO (DOCTYPE , "ar")) AND (LIMIT-TO (LANGUAGE , "English"))	90
Web of Science:<br><br>#1 Cadmium	TOPIC: ((cadmium))<br>Refined by: DOCUMENT TYPES: (ARTICLE) AND LANGUAGES: (ENGLISH) AND DOCUMENT TYPES: (ARTICLE	121.483
#2<br><br>Cancerogenic	TOPIC: ((Genotoxicity OR " ROS formation " OR "Oxidative DNA " OR "Free radical " OR "Free radical* " OR "superoxide dismutase" OR "redox stress " OR "DNA damage ")))<br>Refined by: LANGUAGES: (ENGLISH) AND DOCUMENT TYPES: (ARTICLE)	307.701
#3 Inflammatory response	TOPIC: ((" Inflammatory response" OR " Inflammatory signalling" OR" NF-kB" OR " pro-inflammatory cytokines "OR " IL-6" OR" IL-8 "))<br>Refined by: LANGUAGES: (ENGLISH) AND DOCUMENT TYPES: (ARTICLE)	208.343
#1 AND #2AND #3	TOPIC: ((cadmium)) AND ((Genotoxicity OR "ROS formation " OR "Oxidative DNA " OR "Free radical " OR "Free radical* " OR "superoxide dismutase" OR "redox stress " OR "DNA damage "))AND ((" Inflammatory	69

	response" OR " Inflammatory signalling" OR" NF-kB" OR " pro-inflammatory cytokines " OR " IL-6" OR" IL-8 ")) Refined by: LANGUAGES: (ENGLISH) AND DOCUMENT TYPES: (ARTICLE)	
The total from the three databases:		214
Number after remove duplication: (By Rayyan)		180

Supplementary Table S2. PICO and inclusion and exclusion criteria for the systematic review.

PICO	Inclusion Criteria	Exclusion Criteria
**Population**	Animals/humans/cells	Plants
**Intervention**	Cadmium	Not related to Cadmium
**Comparators**	Untreated Cd/control<br>Treated Cd/control	No Cd
**Outcomes**	Generation of free radical (ROS) and induction of inflammation	Not related to free radical (ROS) generation and induction of inflammation

Supplementary Table S3. Risk of bias and assessment according to the SYRCLE criteria.

RISK OF BIAS	No	Yes	Unclear
1)Was it stated that the experiment was randomized at any level?	10	0	0
2) Was it stated that the experiment was blinded at any level?	10	0	0
3) Was the allocation sequence adequately generated and applied?	10	0	0
4) Were the groups similar at baseline or were they adjusted for confounders in the analysis?	10	0	0
5) Was the allocation adequately concealed?	0	0	10
6) Were the animals randomly housed during the experiment?	10	0	0
7) Were the caregivers and /or investigators blinded from knowledge which intervention each animal received during the experiment?	0	0	10
8) Were animals selected at random for outcome assessment	10	0	0
9) Was the outcome assessor blinded?	0	0	10
10) Were incomplete outcome data adequately addressed?	10	0	0
11) Was the study apparently free of other problems that could result in high risk of bias?	0	10	0

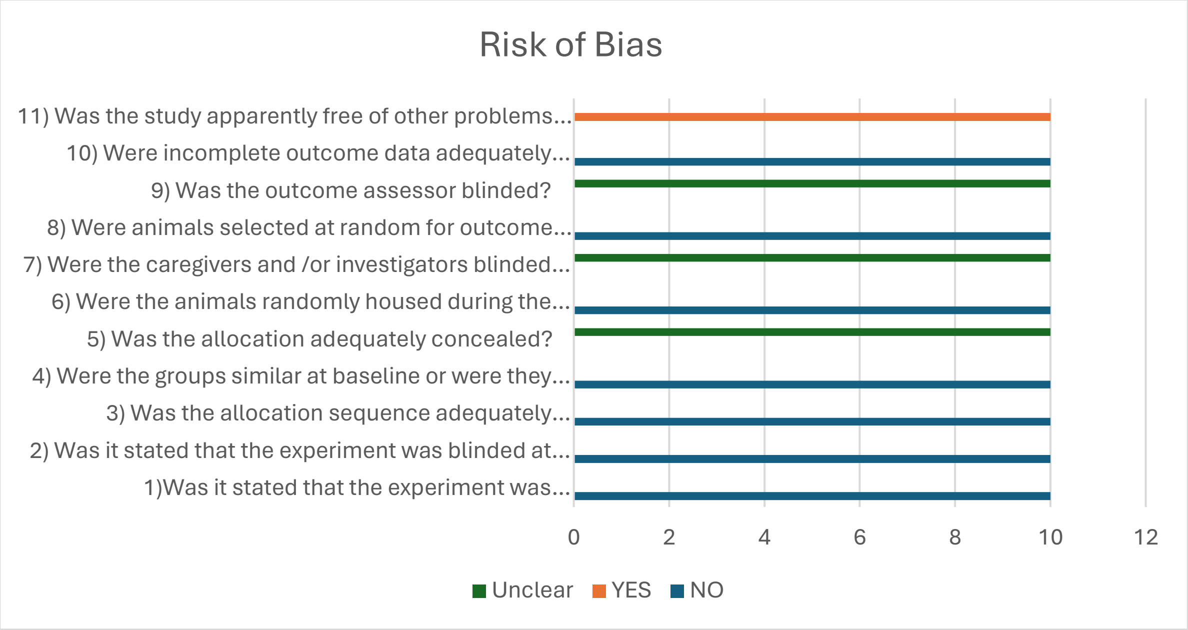

Supplementary Figure S1. Risk of bias and assessment according to the SYRCLE criteria.
